# Dielectric Characteristics of Crosslinked Polyethylene Modified by Grafting Polar-Group Molecules

**DOI:** 10.3390/polym15010231

**Published:** 2023-01-01

**Authors:** Jun-Guo Gao, Li-Wei Liu, Wei-Feng Sun

**Affiliations:** 1 Key Laboratory of Engineering Dielectrics and Its Application, Ministry of Education, School of Electrical and Electronic Engineering, Harbin University of Science and Technology, Harbin 150080, China; 2 School of Electrical and Electronic Engineering, Nanyang Technological University, Singapore 639798, Singapore

**Keywords:** crosslinked polyethylene, polar group molecule, chemical graft modification, charge trap, dielectric breakdown strength

## Abstract

Polar group-modified crosslinked polyethylene (XLPE) materials are developed with a peroxide thermochemical method of individually grafting chloroacetic acid allyl ester (CAAE) and maleic anhydride (MAH) to polyethylene molecular-chains, which are dedicated to ameliorating dielectric characteristics through charge-trapping mechanism. By free radical addition reactions, the CAAE and MAH molecules are successfully grafted to polyethylene molecular chains of XLPE in crosslinking process, as verified by infrared spectroscopy molecular characterizations. Dielectric spectra, electric conductance, and dielectric breakdown strength are tested to evaluate the improved dielectric performances. Charge trap characteristics are investigated by analyzing thermal stimulation depolarization currents in combination with first-principles electronic-structure calculations to reveal the polar-group introduced mechanisms of contributing dipole dielectric polarization, impeding electric conduction, and promoting electrical breakdown field. The grafted polar-group molecules, especially for MAH, can introduce deep-level charge traps in XLPE materials to effectively restrict charge injections and hinder charge carrier transports, which accounts for the significant improvements in electric resistance and dielectric breakdown strength.

## 1. Introduction

Crosslinked polyethylene (XLPE) is an essential industry insulation material due to its pertinent electrical and mechanical performances used for the main insulation of high-voltage (HV) power cables [1]. In China, XLPE-insulated HV direct current (DC) cables have approached the world’s advanced technology of ±320 kV voltage levels [2,3]. In the plastic material-insulated power cables, the XLPE-insulated power cables represent superior merits in electrical performance, power transmission, mechanical flexibility, resistance, heat resistance, thermal stability, etc., which also have the advantages of a simple installation, a large transmission capacity, and the easy processing of accessories [4]. Compared with oil-paper insulated cables, XLPE cables with dry-structured accessories operate in a higher reliability, possess a stronger heat resistance, and lead to a larger transmission capacity, as an essential role in urban power grids [5,6]. With the continuous improvement of power transmission efficiency, it is imperative for XLPE-insulated HV cables to develop onto a higher voltage level. Therefore, it is the inevitable course to develop XLPE-insulated high-voltage cables by improving the operation stability and performances of XLPE main insulation through more advanced material modification technologies.

Linear-structured polyethylene (LPE), as a thermoset material, renders excellent electrical insulation, high chemical stability, non-toxicity, and processing flexibility, which has been widely applied for wire or cable insulation. LPE has a poor thermal resistance and low mechanical strength and is prone to rupture under thermal or mechanical stress [7]. In order to improve the heat resistance, stress cracking resistance, and mechanical properties of LPE, the pristine polyethylene materials with a linear structure are particularly processed into XLPE through polymer crosslinking technologies. XLPE is a thermosetting polymer insulation material developed on the basis of LPE, inheriting the advantages of the high electrical insulation and low-density of LPE whilst acquiring significant improvements in mechanical properties and dielectric performances, which have been widely used in industrial productions of high-voltage and ultra-high-voltage plastic insulated power cables [8]. Nevertheless, XLPE materials are vulnerable to accumulate space charge accumulations under high-voltage electric fields, which engender local electric field distortion and partial discharge, leading to electric trees and even insulation failures [9]. Therefore, effectively improving the dielectric performances of XLPE materials is of great significance for the safe operation of high-voltage power cables.

Polymer dielectric composites for filling inorganic nanoparticles into low-density polyethylene (LDPE) can acquire a significant improvement in the dielectric performances of polyethylene insulation materials by inhibiting space charge accumulations and charge injections [10,11,12,13,14]. For Al_2_O_3_/LDPE nanocomposites, the internal space charge quantity is highly correlated with the nanofiller concentration, which approaches the minimum level at a filling content of 1.0 wt%, implying the optimal balance point of suppressing space charge accumulations with the nanofiller concentration [15]. Nanosilica/XLPE composites with sinyl silane surface-modified silica nanofillers acquire the highest partial discharge inception voltage and dielectric breakdown voltage at 3.0 wt% content rather than other filling contents, in which the peak space charge density is decreased by 57.8% compared to XLPE [16,17]. A small amount of carbon black (CB) filled into XLPE can effectively inhibit the internal space charge accumulations and reduce electric conductivity [18]. The concentration and size of inorganic nanofillers and the states of filler/matrix interfaces are primarily focused on homogenizing space charge distribution, impeding electric conduction, and promoting dielectric breakdown strength and thermodynamic properties of LDPE and XLPE nanocomposites [19,20,21,22]. Additionally, the surface-modified nanosilica by auxiliary crosslinkers has been developed to improve filler dispersivity and polyethylene crosslink degree in nanosilica/XLPE composites, which, meanwhile, acquire substantial improvements in electric-tree aging resistance and dielectric breakdown strength by regulating electric field and introducing deep charge traps [23].

Mechanical and electrical properties of individual polyalkenes can be evidently improved by mixing various polyalkenes to realize the recoverable thermosetting XLPE. The blend material of LDPE intermixed with high-density polyethylene (HDPE) at the mass ratio of 4:1 and cooling rates of 0.5–1.0 K/min represent a significantly higher dielectric breakdown and mechanical strengths than XLPE [24,25]. It is feasible to mix 20 wt% HDPE into ethylene-vinyl acetate copolymer (EVA) or LDPE to significantly increase mechanical toughness, heat-stability, and capability of inhibiting space heterocharge accumulations [26]. The blend method of preparing polymer materials is preferred rather than the crosslinking technique to reduce preparation costs and achieve a lower dielectric loss than XLPE [27]. Metlocene polyethylene (MPE) as a nucleation agent mixed into LDPE has been exploited to successfully diminish spherulites, expedite crystallization nucleation and growth, and homogenize space charge distributions by mitigating electric field distortions at crystal/amorphous phase interfaces [28].

Graft modification is a molecular-level technology of chemically introducing specific functional groups into polymer backbone to promote intrinsic electrical properties of insulation materials used for fabricating HV power cables. Organic compounds with polar groups, such as carbonyl (C=O), used for graft modifications, are competent in reducing charge carrier mobility, ameliorating space charge characteristics, and acting as inhomogeneous nucleation centers to increase polyethylene spherulite density, which account for the holistic improvements in electric-tree resistant performance, insulation strength, and water-tree aging resistance, respectively [29,30,31,32,33]. It is a comprehensive scheme of suppressing the space charge accumulations and enhancing electric resistance of ethylene/α-alkenes copolymers by chemically introducing polar groups, such as ethyl, hydroxyl, nitro, cyanyl, or aromatic ring to improve the electrical performances of power cables under a polar reversal. The successful modification of grafting 4-vinyl oxylphenylacetone to polyethylene molecular chains has been realized during the polyethylene photon-initiated crosslinking reaction, in which it can effectively prevent the functional-group compounds from migrating out of polymer matrix, resulting in a significant improvement in DC dielectric properties of XLPE [34]. By grafting aromatic derivatives, the resistance to electric-tree aging and space charge accumulation in XLPE can be improved significantly at various temperatures [35].

So far, the chemical modifications on polyethylene materials by grafting polar-group compounds to improve the main insulation of high-voltage cables have still not achieved a comprehensive or substantial progress in theoretical research and engineering application. At present, only three articles have reported that chloroacetic acid allyl ester (CAAE) and maleic anhydride (MAH) selected from various polar-group molecules are successful as grafting modifiers to improve DC dielectric performances of XLPE materials [29,30,36], in which the MAH grafting modification adopts the ultraviolet initiation grafting technology, needing further verification for high-voltage cable manufacturing. To date, there are no reports on the alternative current (AC) dielectric performances of CAAE- and MAH-grafted XLPE. Accordingly, the present study focuses on peroxide thermochemical preparations and AC dielectric characteristics of the polar-group grafted XLPE materials and gives a deep insight into the charge-trapping mechanism accounting for electrical insulation strength. The thermal-stimulated depolarization current and electric current density versus the electric field, the complex dielectric function, and the AC dielectric breakdown strength of the grafted materials are tested to elucidate the ameliorated dielectric characteristics, which are also verified by first-principles electronic-structure calculations.

## 2. Experimental and Theoretical Methods

### 2.1. Material Preparation

**Figure 1:**
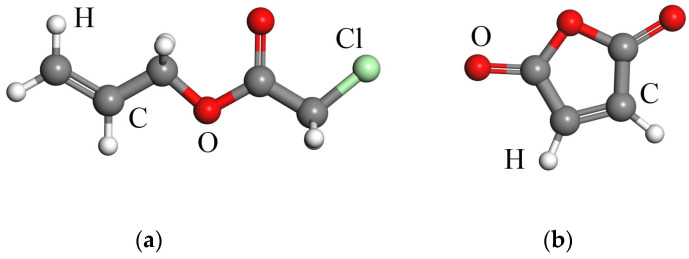
Chemical structural schematics of polar-group molecules for grafting modifications: (**a**) CAAE; (**b**) MAH.

Chloroacetate (CAAE) and maleic anhydride (MAH), which contain carbonyl (C=O) and an unsaturated double-bond (-C=C-), are selected as two representative polar-group compounds for chemical graft modifications, as shown by their molecular structures in Figure 1. In CAAE molecule, both C=O and the carbon-bonded chlorine atom possess a high polarity to capture the charge carriers transporting through polyethylene matrix under high electric fields. In MAH molecule, in addition to the two C=O groups acting as charge-trapping centers, the -C=C- favors grafting MAH to polyethylene molecular chains without self-aggregation.

Together with 2 wt% dicumyl peroxide (DCP, Nobel Company Ltd., Aksu, China) and 0.3 wt% pentaerythritol ester antioxidant (Irganox1010, BASF SE., Ludwigshafen, Germany), the CAAE or MAH (Ruierfeng Chemical Co. Ltd., Guangzhou, China) compound is mixed individually by 0.5, 1.0, and 1.5 wt% contents into low-density polyethylene (LDPE, LD200GH, Sinopec Company Ltd., Beijing, China) at 110 °C and a rotation rate of 40 rpm for 20 min of a melting blend, as implemented in a Torque Rheometer (RM200C, Hapro Company Ltd., Harbin, China). For chemical crosslinking/grafting reactions, the obtained blend of uniformly mixed raw materials is pressed for molding at 110 °C under the increased pressure from 1 atm to 15 MPa by the rate of 1 MPa/min in a plate vulcanizer and, subsequently, further heated-up by the rate of 5 °C/min to 175 °C, persisting for 35 min. Eventually, the crosslinked and grafted polyethylene material is cooled down to room temperature and compressed into film samples, which are then hot-degassed under a short circuit in a vacuum-drying oven at 80 °C for 48 h to eliminate residual small molecules and relax thermal stresses.

### 2.2. Characterization and Test

Infrared absorption spectra of film samples of 0.3 mm thickness are tested in a wavenumber range of 500–4000 cm^−1^ with a resolution of 2 cm^−1^ for the raw material blends before and after the hot-degas treatment, and for the grafted XLPE, after the hot-degas treatment, as implemented in a Fourier-transform infrared (FTIR) spectrometer (FT/IR-6100, Jiasco Trading Co., Ltd., Shenyang, China). The infrared absorption peaks from the characteristic chemical groups before and after crosslinking/grafting reactions are identified and compared to verify chemical graft fulfillment.

Electric conductance is tested by measuring the electric current density as a function of electric field strength at room temperature, as implemented in a standard three-electrode system. The prepared materials are fabricated into circular film samples of 50 mm diameter and 200 ± 10 μm thickness, which are evaporated by aluminum electrodes on both sides. The annular protective electrode with inner and outer diameters of 55 mm and 75 mm, respectively, encircles the disc measuring electrode of 50 mm diameter on one side of film samples, and the circular high-voltage electrode of 80 mm diameter resides on the other side. The stable conductance current is measured after applying DC voltage for 60 min at each point of the testing electric field strength, which is increased by a step-up of 5 kV/mm in range of 5~45 kV/mm.

Thermal stimulation depolarization currents, as a function of temperature, are tested to analyze the energetic distributions of charge traps, as implemented in a temperature-controlled dual-electrode system (TSDC, Harbin University of Science and Technology, Harbin, China). The tested film samples of 100 ± 10 μm thickness are polarized by applying an electric field of 40 kV/mm for 30 min at 50 °C and then promptly cooled down to −50 °C, persisting for 5 min in liquid nitrogen. Depolarization currents are continuously measured by short-circuiting samples and by raising the testing temperature from −50 to 170 °C, with a heating rate of 3 °C/min.

Dielectric frequency spectra of XLPE-based samples are tested at room temperature by applying an AC electric field of 100 kV/mm for 40 min at each frequency point with the testing frequency being increased by a step-up of 1 Hz in range of 1~10^6^ Hz, as implemented in a wide-frequency dielectric spectrometer (Alpha-A, Novocontrol Co., Ltd., Montabaur, Germany) equipped with a spectrum analyzer (N9320B, Agilent Technologies Co., Ltd., Santa Clara, CA, USA).

Dielectric breakdown strength is evaluated by measuring AC breakdown fields at room temperature on circular film samples of 70 mm diameter and 100 ± 10 μm thickness, in which the asymmetric columnar electrodes of 20 mm and 60 mm diameters are used for high-voltage and ground electrodes, respectively. At the end of continuously increasing electric field at rate of 4 kV/s, the maximum voltage automatically recorded just before dielectric breakdown occurred is granted as the breakdown voltage to calculate breakdown field strength.

### 2.3. Molecular Simulation

Polyethylene-graft molecular models of 20 polymerization degrees in carbon backbone being chemically grafted by one CAAE or MAH molecule are initially constructed according to the randomly distributed torsion and rotational isomeric state methods [37]. In first-principles calculations, the initial polymer configurations are geometrically optimized under the conjugated gradient algorithm of energy functional minimization to attain atomic structure relaxation under the convergence tolerance of energy change, atom force, and atom displacement being less than 1.0 × 10^−5^ eV/atom, 0.01 eV/Å, and 0.001 Å, respectively. Molecular orbitals (eigen electron-states) in the energetic representation (density of energetic states, DOS) are calculated to investigate the band-edge features and electronic bound states in band-gaps, which account for carrier mobility and charge traps, respectively. First-principles calculations are performed with all-electron numerical atom-orbital schemes, as implemented by DMol3 module of Materials Studio 2020 package (Accelrys Inc., Materials Studio version 2020.08, San Diego, CA, USA).

## 3. Results and Discussion

### 3.1. Infrared Spectroscopy

**Figure 2:**
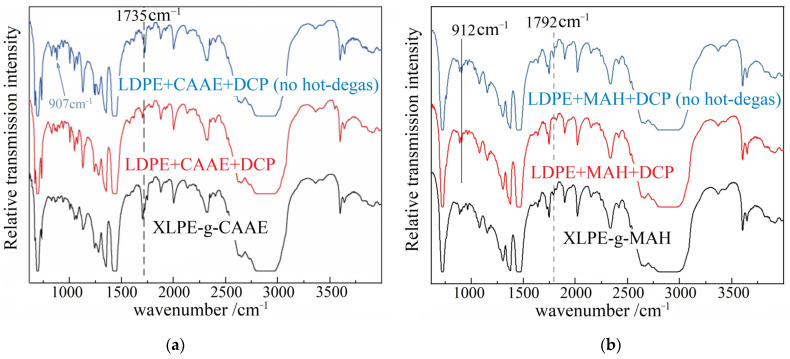
FTIR spectra of the raw material blends (without crosslink and graft) before and after vacuum hot-degas (blue and red curves) and the grafted XLPE (black curves) by (**a**) 1.0 wt% CAAE and (**b**) 1.0 wt% MAH.

Molecular structures of chemical grafts are characterized by the infrared absorption peaks at 907 and 1735 cm^−1,^ respectively, from the stretching vibrations of vinyl (C=C) and ester (C=O) groups in CAAE molecules and at 912 cm^−1^ from MAH molecules, as illustrated in Figure 2. Compared with the raw material blends, in which both the C=C 907 cm^−1^ and C=O 1735 cm^−1^ peaks disappear after the hot-degas treatment in vacuum, the XLPE grafted with 1.0 wt% CAAE (XLPE-g-1.0 wt%CAAE) through the polyethylene crosslinking process merely retains C=O peak, implying the successful graft of CAAE molecules to polyethylene molecular-chains. As similar, the absorption peak at 912 cm^−1^ from the double chemical bonds in MAH molecules vanishes in the infrared spectrum of 1.0 wt% MAH-grafted XLPE (XLPE-g-1.0wt%MAH), which however represents the newly arising peak at 1792 cm^−1^ of carbonyl groups produced by grafting reaction in polyethylene crosslinking process, as a substantial manifestation of MAH graft to XLPE. The identical sink and emergence of characteristic infrared peaks are also presented by the other modified XLPE with 0.5 wt% or 1.5 wt% graft content. It is demonstrated by FTIR spectroscopy that the chemical grafts of CAAE and MAH to XLPE have been realized.

### 3.2. Dielectric Frequency Spectra

**Figure 3:**
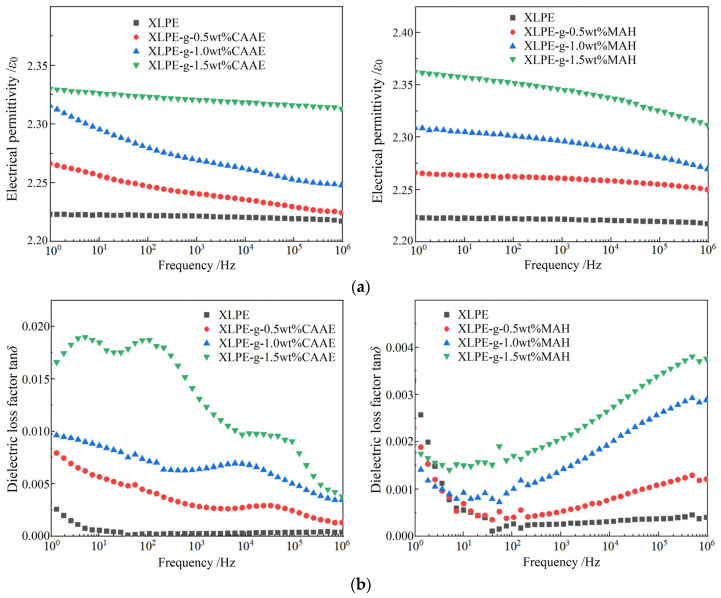
Dielectric spectra of XLPE-g-CAAE (**left panels**) and XLPE-g-MAH (**right panels**) in comparison to XLPE: (**a**) relative electrical permittivity; (**b**) dielectric loss factor tan*δ*.

Although the three published papers have reported DC dielectric performances of CAAE and MAH graft-modified XLPE, no reports have been so far presented for dielectric frequency spectra and AC insulation strength of XLPE modified by CAAE and MAH grafting [29,30,36]. Therefore, the complex dielectric function and AC dielectric breakdown strength of the CAAE- and MAH-grafted XLPE materials are tested and analyzed. Compared with XLPE benchmark, both the relative dielectric permittivity and dielectric loss factor of XLPE-g-CAAE and XLPE-g-MAH are notably higher and increase with the increasing graft content, as shown in Figure 3, which is attributed to the fact that the multiple polar groups in the grafted CAAE and MAH are actual dipoles for undergoing orientation polarization under AC electric fields. As the electric field frequency increases, it is more difficult for these dipoles to be completely polarized in one orientation by keeping up with the alternation of external electric field, resulting in the dielectric permittivity abatement and dielectric loss exacerbation. In the relatively lower frequency region, the dielectric loss is dominantly derived from the resistance to transient electric conduction of oscillation carriers (merely a part kind of charge carriers for electric conductance under DC electric field), which are not derived from the electric dielectric orientations of polar-group dipoles on the grafted CAAE and MAH, as indicated by the declining loss factor with increasing frequency; in the relatively higher frequency region, the dipole orientation polarization gradually fails to keep up with the applied AC electric field so that relaxation polarization loss increases significantly and exceeds conductance loss, as manifested by the higher dielectric loss factor of the graft-modified XLPE than that of the XLPE benchmark, which is intensified with increasing graft content.

The XLPE-g-0.5wt%CAAE and XLPE-g-1.0wt%MAH represent a lower dielectric loss factor than 0.001 in 50~50,000 Hz frequency range. The dielectric loss factors of XLPE-g-1.0wt%CAAE and XLPE-g-1.5wt%MAH maintain lower than 0.001 at 50 Hz but exceed 0.001 in >50 Hz frequency range. The XLPE-g-1.5wt%CAAE does not meet the requirement of AC cable insulation materials due to its higher dielectric loss factor than 0.001 at 50 Hz. Multiple polar-groups of CAAE and MAH grafted on polyethylene molecular-chains could be oriented by AC electric field and, thus, contribute significantly to dielectric polarization, leading to a notable increment for both electrical permittivity and tan*δ*, which are more sensitive in relatively lower and higher frequency regions for CAAE and MAH grafted XLPE, respectively, due to the much longer dipole distance (the separations between the positive and negative charge centers) of CAAE than that of MAH. It is thus suggested that 1.0 wt% MAH graft modification on XLPE maintains the dielectric loss factor’s lower critical value of 0.001 and will achieve the maximized improvement in dielectric performances of XLPE. 

### 3.3. Dielectric Breakdown Strength

**Figure 4:**
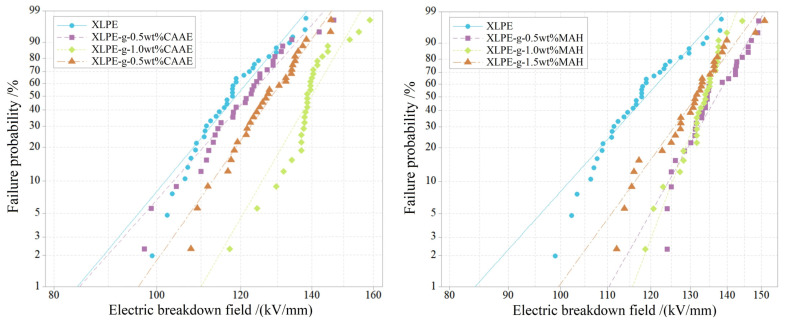
Electric breakdown fields in 2-parameter Weibull statistics for XLPE-g-CAAE (**left panel**) and XLPE-g-MAH (**right panel**) in comparison to XLPE.

The measured AC electric breakdown fields are fitted by two-parameter Weibull statistics to evaluate dielectric breakdown strength (DBS), as shown in Figure 4. The Weibull scale parameter, *E*_b_ (characteristic breakdown field), and the shape parameter, *β* (breakdown resistance stability), are listed in Table 1. For the graft-modified XLPE, the *E*_b_ approaches the maximum at 1.0 wt% graft content. The *β* of XLPE-g-CAAE is higher than that of XLPE benchmark at >0.5 wt% graft content and approaches the maximum at 1.0 wt% graft content; the *β* of XLPE-g-MAH is comprehensively higher than that of XLPE benchmark, and it monotonously increases with graft content. Since the dielectric breakdown under AC electric field is dominantly derived from the thermal breakdown caused by dielectric loss, the DBS of XLPE-g-1.5wt%CAAE with a greater dielectric loss is lower than that of XLPE-g-1.0wt%CAAE with a lower dielectric loss. Meanwhile, the excessive graft content of CAAE or MAH (1.5 wt%) is not preferable due to the substantial aggregation of electron collision ionization or even the dissociation under the high electric field, which however increases local electric field distortions and carrier concentrations, resulting in a higher partial discharge probability, as indicated by the reduced DBS compared with 1.0 wt% graft content.

Compared with other graft contents, the 1.0 wt% graft modifications on XLPE present the most acceptable dielectric polarization characteristics and the highest dielectric breakdown resistance. Therefore, in the following two sections of electric conductance and thermal stimulation depolarization current, the XLPE-g-1.0wt%CAAE and XLPE-g-1.0wt%MAH (as two paradigms of the graft-modified XLPE, abbreviated by XLPE-g-CAAE and XLPE-g-MAH in following sections) are specifically focused on elucidating the underlying mechanism of improving dielectric performances of XLPE by polar-group graft modifications.

### 3.4. Electric Conductivity

**Figure 5:**
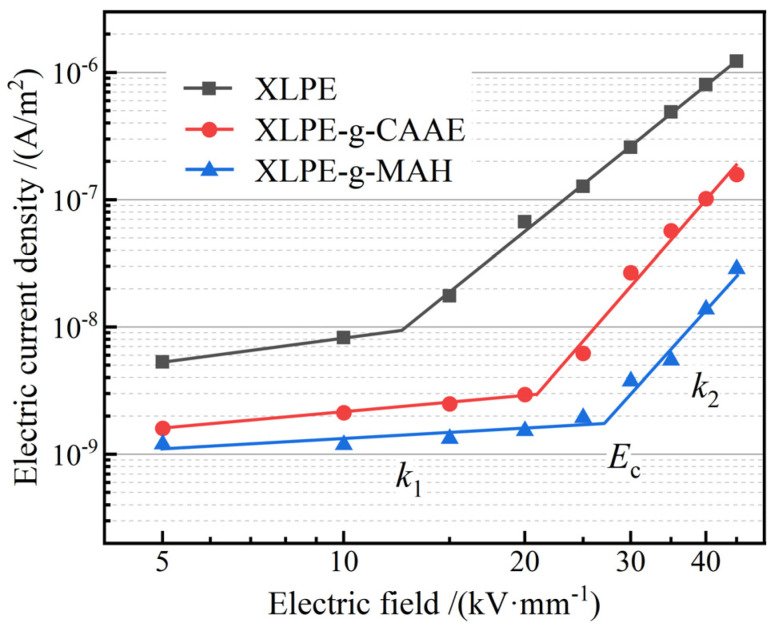
Electric conduction characteristics of *J*–*E* profiles in double logarithm coordinates for XLPE and its modified materials by individually grafting CAAE and MAH.

Characteristic profiles of electric current density versus electric field density (*J*–*E* curves) of XLPE and its graft-modified materials in double-logarithmic coordinates represent a two-stage linearity, as shown in Figure 5. The linearly fitted slopes, *k*_1_ and *k*_2_, are called nonlinear coefficients, and the position of crossing point by *k*_1_ and *k*_2_ lines is called critical electric field, *E*_c_, as shown by the fitted values in Table 2, which distinguishes the transition from Schottky-injected conduction to space charge-limited conduction. The electric current density of XLPE has been remarkably abated to 15 times lower after grafting CAAE or MAH. Meanwhile, the critical electric field of the grafted XLPE is evidently higher than that of XLPE benchmark and even doubled for XLPE-g-MAH. In contrast, the electric conductivity of the MAH-grafted XLPE material prepared by ultraviolet-initiated grafting technology is slightly lower than that of the peroxide thermochemically grafted XLPE material in this study [30]. The peroxide thermochemical grafting method will cause a small amount of halfway reactions and residual-grafting initiators existing in the grafted XLPE, which will provide additional charge carriers under electron thermal excitation, resulting in a slightly higher electric conductivity compared to ultraviolet initiation grafting technology, but still meeting the requirement of cable manufacturing for XLPE insulation.

Under the electric field lower than *E*_c_, the *k*_1_ is less than 1, which complies with Ohm’s law, implying that the charge carriers are mainly rendered by Schottky injections from the electrodes. When the electric field is increased higher than *E*_c_, the space charges begin to accumulate inside the tested film material, where the electric field dominantly relies on space charge distribution and charge carrier density, leading to a nonlinear profile of electric current density versus the applied electric field, as indicated by the remarkably higher value of *k*_2_ than *k*_1_. According to space-charge-limited conduction theory, the electric conduction current follows Child’s law [38]:

(1) $$J=\frac{9\mu \varepsilon U^{2}}{8d^{3}}$$

where *μ* denotes charge carrier mobility, *ε* is electrical permittivity, *U* indicates the applied voltage, and *d* represents film sample thickness. Child’s law describes electric conduction in the ideal condition without any charge traps or when all the charge traps have been occupied. In contrast, all the *k*_2_ are obviously higher than 2, as shown in Figure 5 and Table 2, indicating substantial contributions from charge carrier trapping and detrapping to electric conduction. It is noted that both XLPE-g-CAAE and XLPE-g-MAH present an evidently higher *k*_2_ than XLPE benchmark, identifying the considerable charge traps introduced by the chemical grafts of CAAE and MAH. The graft-introduced charge traps residing on a deeper energy level can retain the validness of capturing charge carriers at a higher temperature rather than releasing charge carriers by thermal excitation. This trapping mechanism leads to the electric conductivity reduction due to the trapped charges scattering with charge carriers, and results in the critical electric field improvement due to the increased Schottky injection barrier by forming electrostatic shielding (Coulomb force) layers near electrodes. These results of electric conductivity are highly consistent with dielectric breakdown experiments and provide a reliable demonstration that the significant DBS improvement of XLPE by these graft modifications should be attributed to the considerable deep charge traps introduced by the grafted CAAE or MAH molecules. 

### 3.5. Charge Trap Characteristics

**Figure 6:**
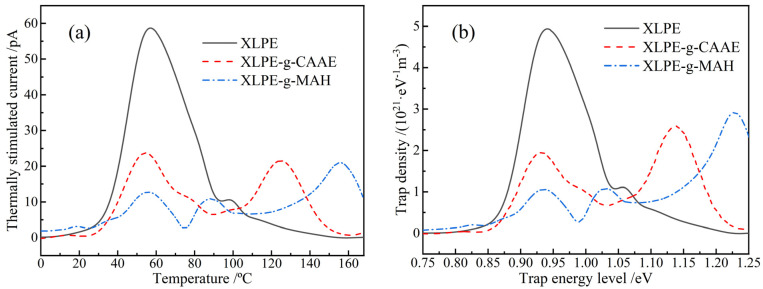
(**a**) TSDC temperature spectra and (**b**) trap level distributions of XLPE benchmark and graft-modified XLPE.

Thermal stimulation depolarization current (TSDC), as an effective characterization of exploring trap level distributions in solid dielectrics, can reveal intrinsic attributes of the charge-trapping and scattering processes in polymer insulation materials. The charge trap characteristics under the present graft modifications are shown by TSDC in Figure 6a. The general TSDC peak at 57 °C is generated from the detrapping charges that have been captured in intrinsic charge traps of structural defects in polyethylene materials. Although there are some relatively deeper traps (such as antioxidant-introduced chemical traps) in XLPE benchmark, which majorly reside on crystal/amorphous phase interfaces, their densities are not adequate to capture enough charge carriers for forming an electrostatic shielding layer near electrodes and are thus incapable of excluding the charge injections from electrodes. The characteristic TSDC peaks evidently appearing at >100 °C temperatures for the graft-modified XLPE identify the sufficient amount of deep traps introduced by grafting CAAE or MAH, which accounts for the significant improvements in electric resistance and DBS under trapping mechanism. The depth of trap level determines the upper-temperature limit for maintaining an effective trap mechanism. At temperatures higher than this temperature’s upper limit, the charge traps are invalid for capturing charge carriers due to the fact that the captured charges will be thermally excited by atomic vibrations which comply with Boltzmann statistics.

The trap level distributions are derived from TSDC spectra, according to the thermal excitation current theory [39], as shown in Figure 6b. The trap density peaks at 0.95 eV and 1.04 eV correspond to the thermally excited releases of the trapped charges in XLPE intrinsic traps and antioxidant chemical traps, respectively. Since the grafted polar-group molecules introduce a large number of deeper-level traps, and charge carriers prefer to fall into deeper traps rather than shallower ones, the XLPE intrinsic TSDC peak intensity is significantly reduced. Compared with the introduced deep trap of 1.12 eV in XLPE-g-CAAE, the graft of MAH is preferable for acquiring deeper traps of 1.23 eV. In comparison, the XLPE-g-MAH prepared by ultraviolet initiation grafting technology can acquire deep charge traps at a depth level of 1.25 eV, which is very close to the 1.23 eV fulfilled by thermochemical method in the present study [30]. Therefore, the CAAE- and MAH-grafted XLPE materials prepared by thermochemical method can effectively introduce deeper traps than the intrinsic charge traps derived from polymer structural defects to inhibit charge injections from electrodes and impede charge carrier transports even at 150 °C.

**Figure 7:**
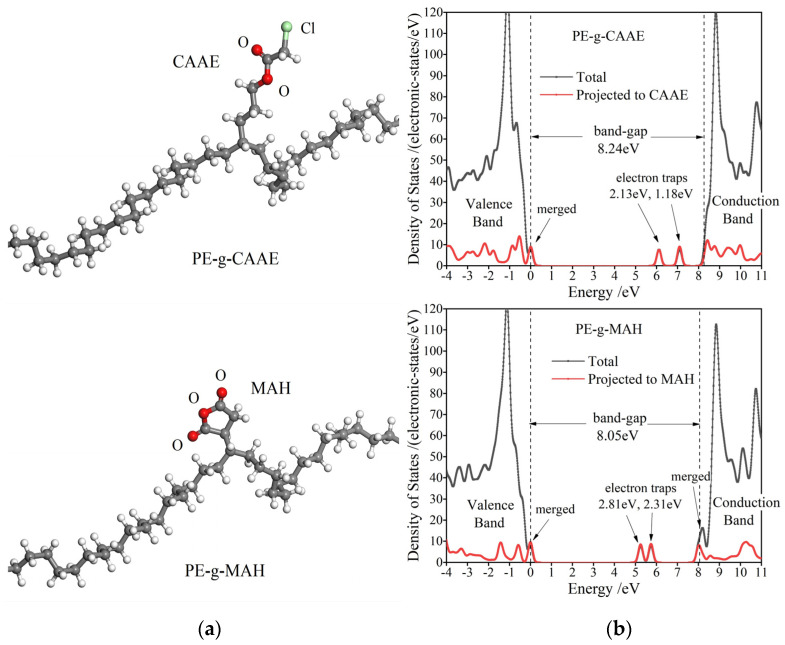
(**a**) Molecular structures after geometry optimization and (**b**) PDOS (Gaussian smearing 0.1 eV) of the polyethylene macro-molecules grafted individually with CAAE and MAH, which are annotated by PE-g-CAAE (**top panels**) and PE-g-MAH (**bottom panels**), respectively, with the highest occupied molecular orbital being referenced as energy zero point.

According to all-electron atomic-orbit first-principles schemes, the polyethylene (PE) grafted with CAAE and MAH molecules are modeled and calculated for electronic properties. Projected density of states (PDOS) is exploited to elucidate the electronic bound states introduced by the grafted polar-group molecules in PE band-gap, which accounts for energy levels and densities of hole traps (the occupied bound states) and electron traps (the unoccupied bound states). The molecular models of the PE grafted with CAAE and MAH (PE-g-CAAE and PE-g-MAH) and their PDOS are shown in Figure 7, and the derived charge trap levels are listed in Table 3. It should be noted that the two molecular models present an identical band-gap of ~8 eV, which agrees well with the experimental result of XLPE material, but they incorporate no electronic bound states derived from intrinsic structural defects of XLPE material as represented in the molecular-level models of multiple-molecule-assembled condensed matters. Both the grafted CAAE and MAH introduce electronic bound states near PE electron band-edges, which are almost degenerated with band-edge levels to be merged into conduction or valence band and hereby become the new band-edges, resulting in a slightly narrower band-gap. Two unoccupied electronic bound states arising below conduction band minimum (CBM) in both PE-g-CAAE and PE-g-MAH models demonstrate, in theory, that deeper electron traps can be introduced by grafting CAAE or MAH into XLPE material.

TSDC analyses and first-principles calculations verify that the charge traps in XLPE-g-MAH are deeper than those in XLPE-g-CAAE, consistent with the electric conductance and AC breakdown tests. The two adjacent carbonyl groups of MAH are coupled to produce the energetic splitting of their two molecular orbitals, with the energy levels residing just below CBM in the band-gap of background PE, which will act as two deeper-level traps to capture and scatter electron carriers at CBM. From Boltzmann thermal excitation theory, it is estimated that the >2.3 eV deep traps introduced by grafting MAH graft and the 2.13 eV introduced by grafting CAAE can be identified by the TSDC only at a test temperature above 300 °C, which ought to be confirmed in the future followed research.

## 4. Conclusions

Thermochemical modifications of grafting organic small molecules with specific polar groups (CAAE and MAH) on the dielectric polarization behavior, carrier transport, and insulation strength of XLPE were analyzed by testing the dielectric spectra, electric conductance, and dielectric breakdown at ambient temperatures. The thermal stimulation depolarization currents, in combination with first-principle electronic structure calculations, were employed to elucidate the intrinsic mechanism of improving the dielectric characteristics from these chemical graft modifications.

The chemical modifications of grafting CAAE and MAH on the XLPE material can introduce deep charge traps in 1.12 eV and 1.23 eV depths, respectively, which will effectively improve the AC electric breakdown resistance of the XLPE material by inhibiting the charge injections from the electrode and hindering the charge carrier transport at temperatures approaching 150 °C. Compared with ultraviolet initiation grafting technology, the MAH-grafted XLPE prepared by conventional thermochemistry shows a slightly higher conductivity, whilst it is significantly lower than that of the pure XLPE material. The grafted CAAE and MAH molecules render additional molecular orientation polarization, leading to the increments of dielectric permittivity and dielectric loss of the XLPE, which will be exacerbated by increasing the graft content. Due to the much longer dipole distance of CAAE than that of MAH, the polar group orientation polarization, which causes an evident increment of both the electrical permittivity and dielectric loss, is more evident in the relatively lower and higher frequency regions for the CAAE- and MAH- grafted XLPE, respectively. The 1.0 wt% graft content of CAAE or MAH is preferable for efficiently improving the dielectric performances of the XLPE. The grafted CAAE or MAH introduces deeper charge traps in the XLPE, as manifested by the reduced Schottky electric conductance and the retarded dielectric breakdown process, compared with the XLPE benchmark. In comparison to the XLPE-g-CAAE, the trap energy levels in the XLPE-g-MAH are deeper and more effective for improving XLPE dielectric performances.

## Figures and Tables

**Table 1:** Characteristic breakdown field of XLPE-g-CAAE and XLPE-g-MAH compared to XLPE.

Material	*E*_b_ /(kV/mm)	*β*	Material	*E*_b_ /(kV/mm)	*β*
XLPE	122.3	12.30			
XLPE-g-0.5wt%CAAE	126.0	11.54	XLPE-g-0.5wt%MAH	134.7	15.24
XLPE-g-1.0wt%CAAE	142.8	17.62	XLPE-g-1.0wt%MAH	139.6	19.50
XLPE-g-1.5wt%CAAE	131.6	14.61	XLPE-g-1.5wt%MAH	135.4	29.17

**Table 2:** Critical electric fields and nonlinear coefficients of logarithmic *J*–*E* characteristics at room temperature.

Material	*E*_c_ /(kV/mm)	*k*_1_	*k*_2_
XLPE	12.54	0.64	3.80
XLPE-g-CAAE	21.15	0.43	5.41
XLPE-g-MAH	27.14	0.27	5.15

**Table 3:** Energy levels of bound electron-states introduced by grafting polar-group molecules, in reference to the energy level of CBM, as trap level depths.

Molecular Model	Band-gap /eV	Electron Trap Level /eV
PE-g-CAAE	8.24	1.18, 2.13
PE-g-MAH	8.05	2.31, 2.81

## Data Availability

Theoretical and experimental results are available from correspondence with the author.

## References

[1] He J., Peng L., Zhou Y. (2017). Research progress of environment-friendly HVDC power cable insulation materials. High Volt. Eng..

[2] Alassi A., Bañales S., Ellabban O., Adam G., MacIver C. (2019). HVDC Transmission: Technology Review, Market Trends and Future Outlook. Renew. Sustain. Energy Rev..

[3] Chen G., Hao M., Xu Z.Q., Vaughan A., Cao J.Z., Wang H.T. (2015). Review of High Voltage Direct Current Cables. CSEE J. Power Energy Syst..

[4] Teyssedre G., Laurent C. (2018). Advances in high-field insulating polymeric materials over the past 50 years. IEEE Electr. Insul. Mag..

[5] Zhou Y., Zhao J., Liu R., Chen Z. (2014). Key technical analysis and prospect of high voltage and extra-high voltage power cable. High Volt. Eng..

[6] Pleşa I., Noţingher P.V., Schlögl S., Sumereder C., Muhr M. (2016). Properties of Polymer Composites Used in High-Voltage Applications. Polymers.

[7] Montanari G.C. (2011). Bringing an Insulation to Failure: The Role of Space Charge. IEEE Trans. Dielectr. Electr. Insul..

[8] Ohki Y. (2013). Development of XLPE-insulated cable for high-voltage dc ubmarine transmission line. IEEE Electr. Insul. Mag..

[9] Mazzanti G., Chen G., Fothergill J.C., Hozumi N., Li J., Marzinotto M., Mauseth F., Morshuis P., Reed C., Tzimas A. (2015). A Protocol for Space Charge Measurements in Full-size HVDC Extruded Cables. IEEE Trans. Dielectr. Electr. Insul..

[10] Kubota T., Takahashi Y., Sakuma S., Watanabe M., Kanaoka M., Yamanouchi H. (1994). Development of 500-kV XLPE cables and accessories for long distance underground transmission line—Part I: Insulation design of cables. IEEE Trans. Power Deliv..

[11] Kubota T., Takahashi Y., Hasegawa T., Noda H., Yamaguchi M., Tan M. (1994). Development of 500-kV XLPE cables and accessories for long distance underground transmission lines—Part II: Jointing techniques. IEEE Trans. Power Deliv..

[12] Fukawa N., Kawai T., Okano Y., Sakuma S., Asai S., Kanaoka M., Yamanouchi H. (1996). Development of 500-kV XLPE cables and accessories for long distance underground transmission line. III. Electrical properties of 500-kV cables. IEEE Trans. Power Deliv..

[13] Takeda N., Izumi S., Asari K., Nakatani A., Noda H., Yamaguchi M., Tan M. (1996). Development of 500-kV XLPE cables and accessories for long-distance underground transmission lines. IV. Electrical properties of 500-kV extrusion molded joints. IEEE Trans. Power Deliv..

[14] Kaminaga K., Ichihara M., Jinno M., Fujii O., Fukunaga S., Kobayashi M., Watanabe K. (1996). Development of 500-kV XLPE cables and accessories for long-distance underground transmission lines. V. Long-term performance for 500-kV XLPE cables and joints. IEEE Trans. Power Deliv..

[15] Lau K., Vaughan A., Chen G., Hosier I., Holt A., Ching K. (2014). On the space charge and DC breakdown behavior of polyethylene/silica nanocomposites. IEEE Trans. Dielectr. Electr. Insul..

[16] Sharad P.A., Kumar K.S. (2017). Application of surface-modified XLPE nanocomposites for electrical insulation partial discharge and morphological study. Nanocomposites.

[17] Sharad P.A., Kumar K.S., Ahmad M.H., Piah M.A. (2018). Space charge and conductivity measurement of XLPE nanocomposites for HVDC insulation–permittivity as a nanofiller selection parameter. IET Sci. Meas. Technol..

[18] Chen Q.G., Xi B.G., Zhang J.F., Wang X.Y., Yang H.D. (2020). Dielectric and space charge characteristics of nano-modified liquid silicone rubber for high-voltage DC cable accessories. J. Mater. Sci. Mater. Electron..

[19] Li G.C., Zhou X.G., Li X.J., Wei Y.H., Hao C.C., Li S.T., Lei Q.Q. (2020). DC breakdown characteristics of XLPE/BNNS nanocomposites considering BN nanosheet concentration, space charge and temperature. High Volt..

[20] Pourrahimi A.M., Olsson R.T., Hedenqvist M.S. (2018). The Role of Interfaces in Polyethylene/Metal-Oxide Nanocomposites for Ultrahigh-Voltage Insulating Materials. Adv. Mater..

[21] Sun W.F., Wang X. (2013). Molecular dynamics simulation study of polyimide/copper-nanoparticle composites. Acta Phys. Sin..

[22] Gao J.G., Zhao H., Sun W.F. (2017). Molecular Dynamics Simulation Study of Parallel Orientation Structure and Gas Transport in Graphite-Nanoplatelet/Polyethylene Composites. Mater. Today Commun..

[23] Zhang Y.Q., Yu P.L., Sun W.F., Wang X. (2021). Ameliorated Electrical-Tree Resistant Characteristics of UV-Initiated Cross-Linked Polyethylene Nanocomposites with Surface-Functionalized Nanosilica. Processes.

[24] Green C.D., Vaughan A.S., Stevens G.C., Sutton S.J., Geussens T., Fairhurst M.J. (2013). Recyclable power cable comprising a blend of slow-crystallized polyethylenes. IEEE Trans. Dielectr. Electr. Insul..

[25] Li L., Zhong L., Zhang K., Gao J., Xu M. (2018). Temperature Dependence of Mechanical, Electrical Properties and Crystal Structure of Polyethylene Blends for Cable Insulation. Materials.

[26] Hosier I.L., Vaughan A.S., Swingler S.G. (2010). An investigation of the potential of ethylene vinyl acetate/polyethylene blends for use in recyclable high voltage cable insulation systems. J. Mater. Sci..

[27] Suh K.S., Kim J.Y., Lee C.R., Takada T. (1996). Charge distribution in polyethylene/ethylene vinylacetate laminates and blends. IEEE Trans. Dielectr. Electr. Insul..

[28] Wang X., He H.Q., Tu D.M., Lei C., Du Q.G. (2008). Dielectric Properties and Crystalline Morphology of Low Density Polyethylene Blended with Metallocene Catalyzed Polyethylene. IEEE Trans. Dielectr. Electr. Insul..

[29] Zhao X.D., Sun W.F., Zhao H. (2019). Enhanced Insulation Performances of Crosslinked Polyethylene Modified by Chemically Grafting Chloroacetic Acid Allyl Ester. Polymers.

[30] Zhao H., Xi C., Zhao X.D., Sun W.-F. (2020). Elevated-Temperature Space Charge Characteristics and Trapping Mechanism of Cross-Linked Polyethylene Modified by UV-Initiated Grafting MAH. Molecules.

[31] Fu Y.W., Zhang Y.Q., Sun W.F., Wang X. (2020). Functionalization of Silica Nanoparticles to Improve Crosslinking Degree, Insulation Performance and Space Charge Characteristics of UV-initiated XLPE. Molecules.

[32] Chen J.Q., Wang X., Sun W.F., Zhao H. (2020). Water-Tree Resistability of UV-XLPE from Hydrophilicity of Auxiliary Crosslinkers. Molecules.

[33] Zheng X.X., Pan Y.C., Sun W.F. (2022). Water-Tree Characteristics and Its Mechanical Mechanism of Crosslinked Polyethylene Grafted with Polar-Group Molecules. Int. J. Mol. Sci..

[34] Dong W., Wang X., Tian B., Liu Y., Jiang Z., Li Z., Zhou W. (2019). Use of Grafted Voltage Stabilizer to Enhance Dielectric Strength of Cross-Linked Polyethylene. Polymers.

[35] Li C.Y., Zhao H., Han B.Z., Zhang H., Zhang C.C., Ai Y. (2018). Effect of Voltage Stabilizer on the DC Insulation Properties of XLPE. Proc. Chin. Soc. Electr. Eng..

[36] Zhao X.D., Zhao H., Sun W.F. (2020). Significantly Improved Electrical Properties of Crosslinked Polyethylene Modified by UV-Initiated Grafting MAH. Polymers.

[37] Rigby D., Roe R.J. (1988). Molecular Dynamics Simulation of Polymer Liquid and Glass. II. Short Range Order and Orientation Correlation. J. Chem. Phys..

[38] Yang J.M., Wang X., Han B.Z., Zhao H. (2014). DC conductivity characteristic of LDPE nanocomposite and its effect on electric field distribution in HVDC cables. Proc. Chin. Soc. Electr. Eng..

[39] Tian F.Q., Bu W.B., Shi L.S., Yang C., Wang Y., Lei Q.Q. (2011). Theory of modified thermally stimulated current and direct determination of trap level distribution. J. Electrost..

